# The economic impact of endo- and ectoparasites in dairy cattle

**DOI:** 10.1186/s13071-025-07064-8

**Published:** 2025-12-02

**Authors:** Tom Strydom, Robert P. Lavan, Siddartha Torres, Juan Carlos Pinilla

**Affiliations:** 1 Reebok, South Africa; 2 Merck Animal Health, 126 E. Lincoln Ave, Rahway, NJ 07065 USA; 3 Veterinary Medicine Program, Agricultural Sciences Research Group-GICA, University of Santander, Bucaramanga, Santander Colombia

**Keywords:** Cattle, Parasites, Dairy, Nematodes, Trematodes, Protozoa, Economics of treatment, Parasiticide, Therapy

## Abstract

**Background:**

Dairy products provide invaluable sustenance for human populations and any factor that impairs dairy production is a threat to our future food supply. Dairy cattle parasitism is a critical and often unrecognized danger that harms cows; threatens producer livelihoods; can reduce food safety, and hurts farm profitability. Specifically, parasites cause illness and death, reduce milk production, slow weight gain, and harm carcass quality. They may cause abortions, transmit serious bacterial diseases, and harm human health. Cattle movement restrictions to prevent parasite spread add to production costs. Two general parasite types are those found internally (endoparasites) or externally to the animal (ectoparasites) and common parasite classes include: nematodes, trematodes, cestodes, protozoans, arachnids, and insects.

**Conclusions:**

This paper reviews global economic and health impacts of dairy cattle parasitism including discussions of testing methods, treatment strategies, and resistance avoidance.

**Graphical Abstract:**

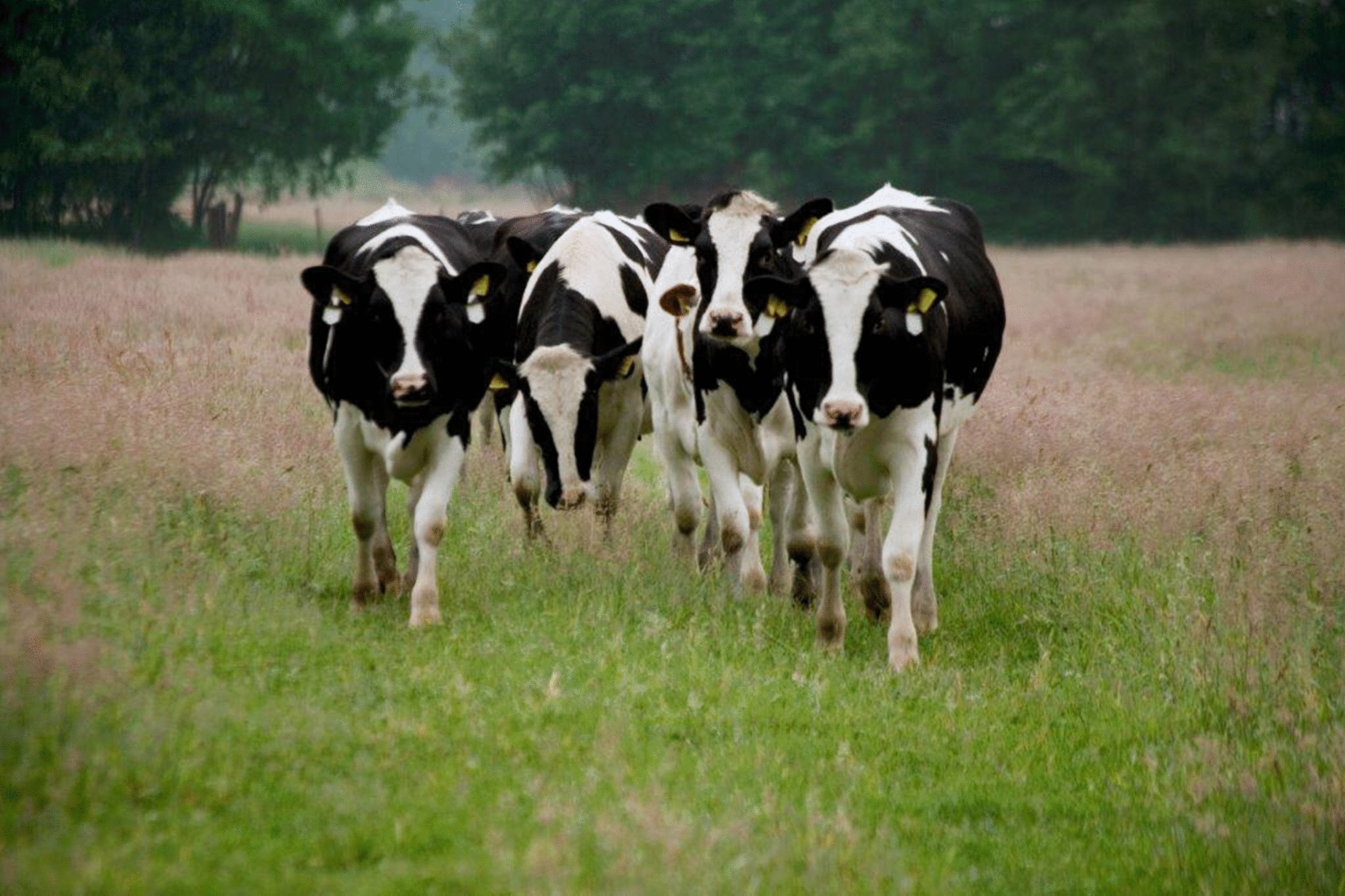

## Background

Dairy products are essential sources of global human nutrition and provide a livelihood for millions of farmers. Dairy production is expected to increase in the future and per capita dairy product consumption is projected to reach 15.2 L by 2032 [1]. The dairy industry is thus vital to future human nutrition and is a vital sector of the global economy that is important for food security, nutrition, and well-being. Parasitism challenges dairy production and this challenge must be met for future world food security [2, 3]. Parasitism harms livestock health in multiple ways: through reduced milk production; slow weight gain; increased carcass loss; illness; death; treatment cost; production option limitations for control measures, abortions; and human food safety risks [2, 4–6]. Parasite control is essential for efficient dairy production and parasite prevention; treatment and control methods may include breeding strategies; animal nutrition; feed sources; land management, diagnostic screening tests; medical treatments; and vaccines [2]. Dairy farm profit margins are often slim, and small changes in production efficiency can make the difference between prosperity and failure [7].

Global climate change facilitate increasing risks of parasite-induced harm; for example, even small mean temperature increases alter parasite life cycles [8], as seen in changes to the abundance, seasonality, and spatial distribution of endemic helminths in UK livestock [9]. Frequency and intensity of UK bovine fasciolosis increased because of increasing temperature and rainfall, and fasciolosis modeling that included climate change considerations found that net average UK dairy farm profit decreased around 12% [10]. These future threats underline the importance of improving parasite control.

This paper introduces important dairy cattle parasites and reviews their economic impact primarily on the basis of health effects [11, 12], primarily in the USA, Europe, and Latin America. Table 1 provides a list of parasites with economic importance for dairy cattle.

**Table 1:** Common parasites with the potential to impact cattle performance

Scientific name	Common name	Likelihood of impact
Internal parasites		
Gastrointestinal nematodes		
*Ostertagia ostertagi*	Brown stomach worm	High
*Trichostrongylus axei* and *T. colubriformis*	Small stomach worm	High
*Cooperia punctata*	Small intestinal roundworm	High
*Oesophagostomum radiatum*	Nodular worm	High
*Haemonchus placei*	Wire worm	High
*Nematodirus helvetianus*	Long-neck worm	High
*Bunostomum phlebotomum*	Cattle hookworm	High
Trematodes		
*Fasciola hepatica*	Liver fluke	High
*Fasciola gigantica*	Giant liver fluke	High
*Paramphistomidae*	Rumen fluke	High
Respiratory nematodes		
*Dictyocaulus viviparus*	Lungworm	Low
Cestodes		
*Moniezia benedeni*	Tapeworm	Moderate
Protozoa		
*Eimeria* spp.	Coccidia	High
*Cryptosporidium* spp.	Coccidia	High
*Babesia bovis* and *B. bigemina*	Hemoparasite	High
*Trypanosoma* spp.	Hemoparasite	Low
*Theileria parva*	Hemoparasite	Low
Rickettsia		
*Anaplasma marginale* and *A. centrale*	Hemoparasite	High
External parasites		
Ticks		
*Rhipicephalus (Boophilus) microplus*	Tropical cattle tick	High
*Rhipicephalus appendiculatus*	Brown ear tick	High
*Amblyomma americanum*	Lone star tick	High
*Amblyomma cajannense*	Cayenne tick	High
Insect		
*Stomoxys calcitrans*	Stable fly	High
*Cochliomyia hominivorax*	New world screwworm fly	High
*Haematobia irritans*	Horn fly	High
*Dermatobia hominis*	Human bot fly	Moderate

Source [15, 16]

### Economic impacts of dairy cattle parasites

This section of the review focuses on available economic research while later sections drill into parasite biology. Parasitism financially harms dairy production because of prolonged heifer rearing, reduced carcass weight, decreased milk yield, impaired fertility, antiparasite treatment purchase and administration, increased labor associated with facility management, food safety, and animal movement restrictions [13].

Global annual costs from helminth infections were estimated at €941 million in dairy cattle [14]. Resistant gastrointestinal (GI) nematode infections to macrocyclic lactones cost a further €11–87 million [14]. Estimated economic burdens resulting from endoparasites in Europe are €1.8 billion. This estimate included several gastrointestinal nematodes, the trematode *Fasciola hepatica*, and the lungworm *Dictyocaulus viviparus*. Factors evaluated were the population proportion managed by grazing, infestation intensity, effects of infestation on productivity [including mortality; anthelmintic treatment costs; and costs of anthelmintic-resistant nematode infestation]. Lost production accounted for 81% of the total and the other 19% went to treatment costs. Treatment with parasite preventives led to an average weight gain increase in grazing calves (315 g/day weight gain) compared with untreated control calves suffering subclinical or clinical parasitism (150 g/day gain) [4]. Milk production increases after GI nematode treatment averaged 0.34–1 L/cow per day [15, 16]. Milk production increased by 1 L/cow per day following anti-trematode treatment 80 and 42 days before expected calving date in Belgium. Also in Belgium, an average increase of 0.97 L in daily milk yield was observed in cows treated with an endectocide at calving [17]. In the Netherlands, milk yield of pastured dairy cattle increased by 1.0 L/cow/day after anthelmintic treatment against GI nematodes [15]. These results indicate a recoverable cost of anthelmintic treatment at around $60 to $70 per cow lactation in grazing dairy herds in temperate climate regions [18]. Dairy farmers in Estonia incurred annual losses from 8% to 9% (US $6.23 per calf/yr) from calf mortality from eimeriosis, an enteric protozoal infestation [19].

Cattle producers in the USA lose over $3 billion annually to GI nematodes [20] with a familiar litany of harm, including suppressed appetites, reduced weight gain and overall growth, and reduced reproductive efficiency [21]. In 2009, 38% of producers reported they do not deworm calves before weaning, and approximately 41% of calves are not dewormed at weaning [22]. Just under 60% of replacement heifers and cows are dewormed once annually or less frequently [22]. Grazing dairy cows in Canada gained an average of 0.97 L/cow/day during the first 6 months of lactation when receiving an endectocide at calving [23]. In addition, exposure to GI nematodes reduced dairy farm productivity, while farms with a low level of GI nematode infection had higher milk production benefits and improved productivity [24]. A daily milk loss of 1.4 L/cow was estimated in Kenyan dairy cattle infested with GI parasites including *Haemonchus* spp., *Trichostrongylus* spp., and *Oesophagostomum* spp. [13]. Adverse effects of lungworm *D. viviparus* on adult dairy cattle in clinical outbreaks include milk production reductions of 4–5 L/cow per day and 1–7% mortality [25, 26].

Economic losses caused by grazing cattle parasitism in Brazil were estimated to reach billions of US dollars annually. This includes GI nematode costs estimated at $7.11 billion; cattle ticks (*Rhipicephalus* / *Boophilus microplus*) at $3.24 billion; horn fly (*Haematobia irritans*) at $2.56 billion; human bot fly (*Dermatobia hominis*) at $0.38 billion; New World screwworm fly (*Cochliomyia hominivorax*) at $0.34 billion; and stable fly (*Stomoxys calcitrans*) at $0.34 billion [27]. Tick-caused losses were estimated at US$968 million per year, with milk production decline responsible for 40–50% of this loss [28]. Brazilian cattle tick infestation caused a reduction of 90.24 L of milk/cow/lactation, while losses nationally were approximately $922.36 million per year [7]. Highly tick-infested Holstein cows produced 2.86 L less milk per day compared with cows with a controlled infestation [29] and losses per cow were 10.6 L over 15-weeks.

Annual economic losses caused by different cattle parasites in Mexico were estimated at: GI nematodes US$445.10 million; *Eimeria* spp. US $23.78 million; *F. hepatica* US$130.91 million; *R. (B.) microplus* US$573.61 million; *H. irritans*US$231.67 million; and *S. calcitrans* US$6.79 million [30]. The total annual economic loss from these cattle parasites was estimated to be US$1.41 billion or US$43.57 per head, while in Brazil a similar group of parasites caused US$65.49 per head losses [30].

The costliest global dairy cattle parasitism is ruminant fasciolosis, a chronic infestation affecting over 600 million animals, causing losses over US$2–3 billion per year [6, 31, 34], Impacts are: liver condemnation [34], decreased milk production, reduced cow fertility [35, 36], and incurred treatment costs [5]. This disease directly affects human health through harm to food production [37].

South American production losses to bovine fasciolosis are estimated to significantly reduce (5.8%) cattle carcass weight, leading to reduced animal value of US$35 per head in Brazil [38]. Ruminant fasciolosis is prevalent in many areas of Europe, and causes an annual loss in dairy cattle in Switzerland of approximately €52 million or €299 per infected animal from reduced milk yield, reduced fertility, and liver condemnation [39]. Belgian dairy herds with *F. hepatica* had an estimated annual loss of milk production over US$83 million [40].

Annual losses from liver condemnation are US$1.94 million in Sudan [41]; US$92.4 million in Uganda [42], and US$0.2 million in Saudi Arabia [42]. Ruminant fasciolosis is widespread in Asia and the Middle East. Total economic loss in Turkey was US$63.03 per animal per year. Losses in Cambodia were US$17.02 million in 2001 [34]. Dairy cow GI parasites caused losses in India at US$1.165 million and milk production increased 4–18% following anthelmintic treatment, despite drug resistance complicating liver fluke control [6].

Improved diagnostic tools are shedding more light on the production impact of fasciolosis in modern cattle systems. Milk production in *F. hepatica*-infected dairy herds decreases by 3–5% [43]. Deleterious effects on heifer reproductive performance are seen with high infection challenges [43]. *F. hepatica* modulates the immune response to coinfections, although these complex interactions depend on which pathogens are infecting animals. Johne’s disease (*Mycobacterium avium* paratuberculosis), bovine viral diarrhea virus, and *Clostridium* spp. infections are diseases that may be more difficult to control in *F. hepatica*-infested cattle [43].

Ectoparasitic ticks cause economic loss from bite discomfort and tick-borne diseases, and threaten about 80% of the world’s cattle. One estimate of annual global economic losses is US$22–30 billion [44] and another is US$13.9–18.7 billion [30]. Total annual losses attributable to tick infestations on Brazilian cattle are US$3.2 million [27]. Losses to Mexican dairy herds were US$68 million in 2013 [30]. Argentinian dairy cattle with the tick transmitted disease Babesiosis suffered 38% mortality and a further 62% losses from disease control costs. Babesiosis and anaplasmosis (another tick-borne disease) control cost Australian cattle producers US$16.9 million annually, with tick bite problems (itching and general discomfort) adding US$6.4 million [45]. Annual losses and control costs for babesiosis and anaplasmosis in Kenya were US$5.1 million; Zimbabwe US$5.4 million, Tanzania US$6.8 million, South Africa US$21.6 million, China US$19.4 million, India US$57.2 million, Indonesia US$3.1 million, and Philippines US$0.6 million [46]. Cattle deaths from *Theileria parva* (tick transmitted East Coast Fever) in Africa in the 2000s cost more than US$300 million [47]. An estimated 40 million African cattle are at risk of *T. parva* infection [47].

The horn fly *Haematobia irritans* is the most important insect pest affecting US cattle [48]. Horn fly-associated cattle weight losses in Brazil and Argentina were estimated at 3.25 kg per cow in 305 days of lactation, and USA dairy farms were estimated to lose 26 L (27.0 kg) milk production per cow per year [30]. The stable fly *Stomoxys calcitrans*, is another economically important global livestock insect pest. USA losses were estimated at US$608 million 20 years ago; and current US national dairy cattle losses are estimated at US$360 million on the basis of interpolation of the earlier figures [49]. Estimated milk production losses in USA and Mexican dairy cattle were 26.2–134.7 L milk per cow per lactation [30].

Tabanids are harmful flies that cause production decreases up to 25% in grazing dairy cows. The painful bites of these flies are very stressful and cause skin damage along with decreased food consumption, reduced milk production, and lower feed utilization efficiency. Animals protected with fly repellents can gain more than 0.1 kg/day compared with unprotected animals, while feed utilization efficiency can decrease 17% with blood loss up to 200 mL per day for unprotected animals during fly season [50].

### Deeper insight into economically important parasites

This section of the paper summarizes health effects of the multiple endo- and ectoparasites responsible for significant dairy production losses [5, 14, 27, 30].

### Endoparasites

#### Nematodes

Gastrointestinal nematodes infesting grazing ruminants are clumped together under the general pathological diagnosis of parasitic gastroenteritis. This complex is a damaging constraint for global cattle production responsible for great economic losses for dairy producers [18, 51, 52]. The GI nematodes have a direct life cycle with cattle infestation during pasture grazing. Eggs are passed in feces and develop in the fecal pat from the first-stage larva (L1) to the infective third stage (L3) over 1–2 weeks under optimal environmental conditions. L3 larvae retain their L2 cuticle which protects from harsh environmental conditions as they migrate from fecal pat to nearby plants. L3 are ingested by cattle and then lose their sheath in the rumen before developing further in abomasal or intestinal mucosa, depending on the parasite species. The larvae molt two further times and then emerge as adults on the mucosal surface around 3 weeks after infection, ready to begin shedding eggs [53]. In temperate areas, parasite eggs are deposited on pasture in spring, and larval pasture contamination builds up over summer until early autumn. In winter and early spring, pasture contamination declines to minimal levels [54]. This cycle allows for calendar-based control to minimize pasture contamination and reduce animal infection. Control includes regular diagnostic testing for parasite prevalence and should include an assessment of treatment resistance. Diagnosis is based on sensitive, precise, accurate, and low-cost copromicroscopic techniques using simple Mini-FLOTAC and Fill-FLOTAC devices [55]. Fecal egg count (FEC) is the most used diagnostic test for many nematode and trematode infections; however, other approaches are becoming available and may be affordable and practical in the near future. For example, serology and milk-based assays (e.g. ELISA) are available for certain parasites such as *Ostertagia* and *Fasciola hepatica* (liver fluke).

Parasitic gastroenteritis is a serious concern for dairy calves leaving the barn/stable and entering pasture in temperate climates, and the parasite burden can quickly overwhelm and kill stabled calves introduced to pasture. The first 4 months on pasture is extremely dangerous for dairy calves. The dangerous GI nematodes encountered in dairy production are *Ostertagia* spp., *Cooperia* spp., and *Haemonchus placei* [56].

Nematode genera most associated with parasitic gastroenteritis are: *Haemonchus*, *Ostertagia*, and *Trichostrongylus* in the abomasum; *Cooperia*, and *Nematodirus* in the small intestine; and *Oesophagostomum* in the large intestine [56]. The most important of these in dairy cattle in temperate regions is *Ostertagia ostertagi*. The high pathogenicity of this parasite results in severe clinical disease even when present in low numbers and the economic importance of this parasite was only appreciated with the availability of effective anthelmintics. [56]. *Haemonchus placei* is another highly pathogenic nematode that causes high mortality, particularly in weaned calves. *Cooperia* spp. are commonly encountered in younger cattle and are generally less clinically significant than *Ostertagia* spp. or *Haemonchus* spp. [56], but in warm, wet conditions, may be present in very large numbers and can become economically and clinically significant [57]. Young cattle with parasitic enteritis generally show poor growth and occasional mortality [58].

Parasitic gastroenteritis in the southern USA subtropical climate is associated with: *Cooperia* spp. (*C*. *oncophora*, *C*. *punctata*, and *C*. *pectinata*), *H. placei* and, *O. ostertagi* [57]. Endoparasites identified by coprological tests and polymerase chain reaction (PCR) assays in weaned beef calves from 24 USA states [59] included 91% with *Cooperia* spp., 79% *Ostertagia* spp., 53% *Haemonchus* spp., 38% *Oesophagostomum* spp., 18% *Nematodirus* spp., 7% *Trichuris* spp., and 3% *Trichostrongylus* spp. In addition, 59.9% had coccidia and 13.7% had cestode eggs. These beef calf data may indicate similar risks in dairy calves. Western European dairy cattle had GI nematode infestation prevalence of 3% in Switzerland [60]; 17% in Germany [61]; 20% in Belgium [62]; 63% in Ireland [63]; and 80% in the Netherlands [64].

*Dictyocaulus viviparus* (lungworm) causes verminous pneumonia, a serious sometimes fatal, respiratory disease of cattle of all ages and a recognized cause of poor health in young grazing stock [65]. Parasitic bronchitis, characterized by immune-mediated inflammation, is seen in dairy cows in response to lungworm larvae invasion [66]. Fenbendazole is used as an extremely safe and efficacious lungworm dewormer, destroying worms within the first 12 h after treatment.

#### Trematodes

Fasciolosis (*Fasciola hepatica* and *Fasciola gigantica*) is an unfortunately neglected zoonotic disease with a global distribution. These parasites infest over 17 million people in more than 70 countries [67, 68]. *F. hepatica* is more prevalent in temperate climates, and is also found in tropical and subtropical countries, including the Middle East (Egypt and Iran), South America (Bolivia, Ecuador and Peru), and Asia. *F. gigantica* causes tropical fasciolosis, and is primarily found in less-developed regions of Asia, Africa, and the Middle East [34, 69]. Global fasciolosis prevalence in ruminants (2000–2015) varied widely: Africa 1.2–91%; South America 3–66.7%; Asia 0.7–69.2%; Australia 26–81%; and Europe 0.12–86%. Prevalence variation may be associated with medical treatment, producer awareness, effective control measures, and climate variations [34]. A 95.5% seroprevalence was reported in Peruvian cattle [70] and a 23.76% prevalence was reported on coprology testing of dairy cattle in Mérida state, Venezuela [71]. In Zulia state, Venezuela, 7.31% *F. hepatica* seropositivity was reported in a herd of native breed cattle [72]. The national *F. hepatica* prevalence in Colombian cattle is 25%, and varies between 25% and 80% [32, 73]. A very high prevalence of 86% is reported in cattle in Argentina, compared with reports of 3–66.7% in Brazil, 5% in Colombia, and 11.4–24.4% in Mexico [34]. *F. hepatica* prevalence in cattle in the USA is reported as 5% [74], although other reports found a prevalence in beef cattle in California of 53% and Florida of 68% [75]. In dairy cattle in Switzerland, the prevalence of infection with *F. hepatica* is over 16% [39].

Liver flukes affect ruminants more frequently than other livestock [76] although other mammals (horses, capybaras, deer, and humans) can be infested and serve as reservoirs [77]. *F. hepatica* and *F. gigantica* have a complex life cycle typical of trematodes, requiring an aquatic snail intermediate host for transmission [78]. These snails are in the large Lymnaeidae family with species on all continents except for the poles [79]. In South America, these snails predominate at 2500–2900 m altitude, including the Andes in Colombia, Ecuador, Venezuela, and Peru [80, 81]. Intermediate fluke stages develop in the snail for several weeks until a larval form called a cercaria emerges and attaches to vegetation that is subsequently ingested by cattle. Once in the animal, juvenile flukes migrate through the peritoneal cavity, penetrate the liver, and migrate to the bile duct, causing obstruction and cholangitis. Liver flukes complete their life cycle in 4.5–6 months, at which time they begin to shed eggs in the feces and contaminate pastures [82]. Main consequences of fasciolosis in dairy cattle are anemia caused by adult flukes feeding on blood; liver damage from fluke migration; metabolic and endocrine dysfunction; and immunomodulation that can alter host responses to other pathogens and lead to more sever disease, particularly with bovine tuberculosis [83].

Fasciola adults are leaf-shaped and live in the bile ducts and gall bladder: *F. hepatica* adults are ~4 cm long and ~1.5 cm wide, while the larger *F. gigantica* are ~7.5 cm in length and ~1.5 cm wide. *Fasciola* spp. are hermaphroditic and capable of self-fertilization; however, cross-fertilization is their most common reproductive strategy, and this contributes to gene polymorphism. Liver flukes live for a long period within the host and produce thousands of eggs per day that are released into the intestine and passed in feces [69].

Rumen fluke infections (*Paramphistomidae*) are trematodes that infect ruminants worldwide. Studies have shown an increase in their prevalence in Europe, particularly the species *Paramphistomum daubneyi*, in recent years. They have a heteroexeneous life cycle that includes snails, usually freshwater snails, as intermediate hosts. In most cases, adult flukes appear to inhabit the rumen without damage, whereas immature flukes can cause hemorrhagic lesion of the mucosal tissues, inducing severe morbidity through loss of protein, reduced milk production and weight, edema, and eventually death [84]. The impact of paramphistomosis on dairy cattle health and production is poorly established. However, negative impacts on milk production and growth have been reported [84]

Diagnostic advances have significantly improved coprological and serological methods to detect infestation. *F. hepatica* diagnosis involves egg detection using coprological techniques, including sedimentation or centrifugation [85]. These techniques have a lower sensitivity with a high false negative percentage [86]. The parasitic load and infection timing affect testing success. Coprological techniques will not detect early infections because of the 10–12-week prepatent period. ELISA serological techniques have a higher sensitivity and specificity through detection of *F. hepatica* antibodies as soon as 14 days after infection [87]. The type of diagnostic test used is important for evaluating and comparing the national prevalence data shown above.

### Ectoparasites

#### Arachnids—ticks and mites

Ticks have a tremendous negative impact on global cattle production because tick burdens affect several growth and metabolic characteristics of growing cattle; effects that are exacerbated by poor nutrition [87]. There are more than 920 tick species known, but only a few are of economic concern to dairy producers [89]. Ticks harm by feeding on cattle blood; through bite injuries and by disease transmission. Ticks become disease transmitters because their complex life cycles involve feeding on different hosts. Juvenile tick life stages including larvae, and nymphs become pathogen infected and subsequently transfer these pathogens to other animals during blood feeding. Ticks transmit pathogens to other ticks horizontally when host feeding; transtadially from one life stage to the next through a molt; or vertically from adult through eggs to larvae [90]. These mechanisms can lead to high proportions of ticks carrying pathogens.

*Rhipicephalus (Boophilus) annulatus * (cattle tick) and *R. (B.) microplus* (southern or tropical cattle tick) are hard ticks that harm dairy livestock production particularly in warmer climates. *R. (B.) microplus* has a two-phase life cycle with parasitic and free-living stages [88]. The parasitic phase lasts approximately 21 days and is the life stage responsible for dairy producer economic losses. Understanding this tick life cycle is very important for implementing effective control measures [90].

*R. (B.) microplus* is the most important global tick parasitizing livestock and can produce heavy tick burdens on cattle in Asia, Australia, and Central and South America [91]. Huge economic losses follow from tick caused blood loss, stress, productivity losses, immune suppression, hide damage, and pathogen transmission [92].

Mites are small arachnids that complete their life cycle on the host and feed on skin and skin secretions. Mite infestation is called acariasis and occurs when these pests multiply and lead to skin inflammation. Severe acariasis is called mange, and mange severity depends on mite biology, the preferred mite environment e.g. skin burrows or hair follicles, and cattle housing conditions. Sarcoptic mange is caused by the skin burrowing mite *Sarcoptes scabiei* var. bovis. This mite is highly contagious, causing intense pruritus and papules, and can spread to people. *Psoroptes ovis* is a nonburrowing mite causing psoroptic mange. This mite pierces skin and ingests wound fluids, and the drying fluids lead to appearance of a thick crust. Exudative dermatitis, alopecia, and intense pruritus characterize psoroptic mange and this infestation can kill untreated calves. Early detection and diagnosis are important for infestation control and prevention transmission to the herd [93].

#### Insects—flies

*Haematobia irritans* (horn fly) is a blood feeding fly closely associated with grazing cattle, mainly in Latin American countries, USA, and Australia. Adult flies congregate on the back and shoulders of cattle, or on the underbelly during daytime heat. Fly feeding irritates cattle and results in production losses. Flies lay eggs in dung pats and these become sites of large larval populations. Several insecticide and repellent formulations are used to control flies and treat infestations, although insecticide resistance is an increasing problem [93].

*Stomoxys calcitrans* (stable fly) is another globally distributed blood feeding cattle fly. This fly feeds on blood from a painful bite on lower body parts. It can be a pest in open pastures or in confined facilities. Larvae grow in moist decaying organic material such as crop residues, lawn clippings, silage, and animal bedding. Property clean-up around cattle-raising areas will suppress local fly population growth. Insecticide treatments help control adult flies and biological control methods are available to augment these treatments [93].

The Tabanidae fly family includes several *Tabanus* spp. commonly called horse flies. Larvae develop in aquatic environments and prey on other insects or feed on decaying organic matter. Adults are most abundant in open areas along the forest edge and only females deliver painful bites to feed on blood. These flies can become mechanical pathogen vectors when they return to feed on other cattle in the herd. Fly traps and insect repellents are used for control; however, large tabanid populations are difficult to control when local conditions favor fly population build up [93].

#### Vector-borne pathogens

Bovine babesiosis (tick fever or piroplasmosis) is caused by a tick-borne intra-erythrocytic apicomplexan hemoprotozoan [94]. This disease likely harms global livestock production much more than is appreciated from current data because of the large number of subclinically infected cattle. There are six *Babesia* species: *B. bigemina*, *B. bovis*, *B. divergens*, *B. major*, *B. occultans*, and *B. argentina* [95]. *B. bovis* and *B. bigemina* are present in Asia, Africa, Australia, Central, South America, and Southern Europe [96], where *Rhipicephalus* spp. ticks are the main vector [46]. General global prevalence estimates are approximately 29% of cattle infected, based on studies on six continents (Asia, Africa, Australia, Europe, North America, and South America) and 62 countries. The highest prevalence of bovine babesiosis was in South America at 64%, followed by Australia at 61%, North America at 52%, Africa at 27%, Europe at 22%, and Asia at 19% [97]. *B. bovis* and *B. bigemina* are the most harmful species and cause major economic impacts for cattle producers. In addition to the medical problems described earlier, bovine babesiosis leads to cattle and product movement restrictions that impair international cattle trade. Medical treatments are available for affected cattle.

Bovine anaplasmosis is a tick-borne disease caused by intracellular *Anaplasma marginale* bacteria [98] responsible for clinical disease outbreaks in dairy cattle around the world, particularly subtropical and tropical regions. Climate change will likely increase vector spread, also facilitated by cattle transportation without careful disease screening [99]. A prevalence of 51.7% was reported in Kansas, USA [100]. *Anaplasma centrale* infections are usually asymptomatic, while *Anaplasma phagocytophilum* and *Anaplasma bovis* infect cattle can cause clinical signs, although not commonly [101]. Iatrogenic *Anaplasma* transmission is possible and instrument hygiene and maintenance is an important preventive action.

*Theileria parva* (East Coast Fever) is a protozoan parasite transmitted by the brown-ear tick, *Rhipicephalus appendiculatus*. This infection is found in many African countries and kills over one million cattle a year. Dairy cows on pastoral systems are particularly affected [47].

Many tick transmitted infections can be diagnosed using Giemsa-stained blood and/or organ smears which offer advantages of lower cost and faster results [46]. Blood films are prepared from blood samples collected from capillaries on the tail tip or ear margins and unfortunately poorly prepared or unsuitable smears can preclude diagnosis. *A. marginale* is more easily found in blood smears during the acute disease phase; however, smears have a low sensitivity in asymptomatic cattle. Serologic antibody detection is possible, although seropositivity may indicate chronic disease and these tests are primarily used in research studies, for epidemiological data, export certification, or to investigate possible vaccine failure. Types of serological tests include complement fixation, capillary agglutination assays, card agglutination, and ELISA [101]. Higher sensitivity may be achieved with newer techniques including PCR assays, in vitro cultures and inoculation into susceptible (splenectomized) calves.

Acute bovine tick-borne bacterial infections may be treated with antibacterial drugs. Antibacterial treatment at the time of vaccination could help by reducing the number of infecting pathogens without inhibiting immune responses [46]. Dairy producers introducing new animals may administer prophylactic antibacterials, although this treatment can be costly, and all antibacterial use should consider the impact on potential pathogen resistance.

Vaccine development is needed for tick-borne disease control [101]. Vaccines are an option for bovine anaplasmosis in some countries, although these need further research and development for prevention of persistent infections and elimination of *A. marginale* reservoirs. Development of vaccines against *Babesia* has been difficult because of the number of antigenic strains in circulation [99, 101].

Acaricide use for tick populations control is essential to reduce the risk of tick transmitted diseases including *Babesia*, *Anaplasma*, and *Theileria*. Effective systemic insecticides can kill ticks before pathogens are transmitted into animals at the time of blood feeding. Acaricides must be considered carefully for lactating cows, respecting all milk withholding requirements and recognizing this cost associated with treatment. An approach used is to treat heifers with acaricides until they give birth.

### Parasite control overview

It is important to raise farmers’ awareness that internal parasites are not readily visible yet can substantially affect animal health, productivity, and affect farmers economics. The veterinarian plays a crucial role in educating farmers on the value of routine fecal examinations and on selecting and administering appropriate anthelmintic treatments. Although veterinarians advise on parasite management where parasiticides require a prescription, in many regions (e.g., Latin America, Africa, Asia, Australia, and the USA) anthelmintics are sold over the counter and farmers often choose products and timing on the basis of empirical, nonevidence-based information. In both contexts, veterinarians play a crucial role in education, raising awareness, and promoting stewardship of parasite control.

Parasite control in adult dairy cattle and in heifer-raising operations can be achieved using techniques developed for grazing beef cattle [57]. Appropriate, timely, and targeted anthelmintic treatment programs combined with pasture management practices including the life cycles of the parasite(s) of interest, breeding strategies for genetic improvement, and introduction of new biotechnological tools and diagnostic techniques provide optimal GI nematode control [18, 102]. To preserve refugia when moving animals between pastures, it is a good practice to administer an effective parasiticide only after animals have been placed on the new pasture. Conversely, when introducing new animals into a herd the priority is to avoid introducing parasites with anthelmintic resistance. New arrivals should therefore be treated with the most effective available parasiticide while in quarantine, and a fecal egg count reduction test (FECRT) should be performed during quarantine to confirm treatment efficacy. Only after a successful FECRT should animals be integrated into the resident pastures. Fecal egg count reduction tests are a simple parasitologist-recommended (American Association of Veterinary Parasitologists) screening method used to verify that administered treatments are effective. A fecal parasite check conducted at the time of antiparasitic treatment and then repeated 14 days later [103] provides fecal egg count monitoring and supplements PCR testing for nematode species in grazing cattle [104].

Effective anthelmintic treatments have been successfully used for nematode control in livestock for more than 50 years. This broad group of anthelmintic treatments includes: benzimidazoles, probenzimidazoles, imidazothiazoles, tetrahydropyrimidines, macrocyclic lactones, salicylanilides, substituted phenols, aminoacetonitrile derivatives, cyclic octadepsipeptides, spiroindoles, and praziquantel. Several yearly strategic treatments with 10% albendazole effectively controlled GI nematodes and *F. gigantica* in grazing dairy farms in the Iringa region of Tanzania [105]. Anthelmintic treatment of recently calved cattle can significantly increase future milk production through nematode control [106]. In the absence of resistance, albendazole is more effective for reducing the helminth parasitic load compared with levamisole treatment. Cattle should be tested for GI parasites by regular fecal examination in addition to deworming [106]. This recommendation is important because dairy producers may administer antiparasitic treatments without knowing the specific parasite risks and the associated health problems [107].

Research has brought updated broader spectra antiparasitics to veterinarians and producers that permitted more targeted activity and improved safety. The macrocyclic lactones, ivermectin for example, are a new group of compounds that dramatically improve nematode control with a wide spectrum. Macrocyclic lactones are available as pour-on or injectable forms [108]. Macrocyclic lactones are highly effective against immature and adult nematodes and have activity against ectoparasites [109]. Four treatments per year with ivermectin resulted in profitable management and control of GI nematodes in growing heifers raised in a semi-intensive grazing system in southern Brazil [110]. Finally, a very new class of products called isoxazolines are becoming commercially available for use in cattle and these are directed primarily at ectoparasite control.

Gastrointestinal nematode resistance to anthelmintic treatment is a concern with worldwide reports and potential impacts for multiple nematode and livestock species. Multi-drug resistance (i.e. resistance to more than one compound class) has been identified in all economically important ruminant GI nematodes, including *Haemonchus placei*, *Cooperia* spp., *O. ostertagi*, *Nematodirus battus*, and *Trichostrongylus colubriformis* [111]. Resistance is seen as treatment failures and potentially occurs following failure to exercise care in the timing and frequency of anthelmintic treatment administration [112]. Affected production facilities may face more difficulty in developing an effective parasite control program. Pastures in the USA Midwest evaluated during grazing season were infested with parasites refractory to treatment with macrocyclic lactones (avermectins, milbemycin), and benzimidazole at label-recommended doses [113]. *H. placei* strains resistant to both drug classes were identified, while *Cooperia* spp.—mainly *C. punctata*—and *H. placei* strains resistant to only macrocyclic lactones were identified. Macrocyclic lactone resistance may be widespread on USA farms because this compound class is used for both endo- and ectoparasites [103].

Concerns about potential treatment resistance are driving changes in GI nematode parasite control recommendations. New approaches for ruminant parasite control emphasize the importance of pretreatment diagnostic screening. Appropriate diagnostic tests are implemented that identify infesting parasite species and evaluate their treatment resistance before selecting and administering an antiparasitic medication. Alternative treatment and management strategies can be implemented for parasites exhibiting macrocyclic lactone resistance in grazing dairy cattle [104]. *Cooperia* spp. are typically a threat to grazing cattle on grass and treatment with both a benzimidazole and a macrocyclic lactone at the beginning and during the middle of the grazing period may provide better *Cooperia* spp. control [16]. Concurrent use of products with different modes of action helps to control nematodes and delay resistance development. A USA recommendation is to administer a macrocyclic lactone formulation for ectoparasite control concurrently with a nonmacrocyclic lactone class endoparasiticide to reduce the risk of macrocyclic lactone resistant endoparasites surviving and infesting other cattle [103]. Concurrent use of macrocyclic lactones with oral levamisole was highly effective for minimizing nematode resistance in southeastern USA farms. Eprinomectin, another avermectin, is used in lactating cattle in the European Union (EU) and other countries because it has no milk withholding but may become associated with resistance in the future.

Treatment resistance in pathogenic species such as *O. ostertagi* is a serious concern for dairy producers [114]. *O. ostertagi* resistance to macrocyclic lactones is reported in dairy calves in North America [56]. An efficacy test before anthelmintic treatment administration will ensure these treatments are providing adequate control of infestations [115]. *C. moncophora* are present on almost every farm in New Zealand and are resistant to macrocyclic lactones and many are also resistant to benzimidazoles [116]. New Zealand farmers continue to use macrocyclic lactones [117], consequently, *C. oncophora* control is failing. The clinical impacts of this parasite may not be evident, but this infestation likely causes detrimental effects on animal performance [54].

European reports of resistance to treatment are more limited, although resistant cattle GI nematodes were documented across 16 European countries. Average farm-level resistance prevalence across Europe ranges from 0–100% for benzimidazole and macrocyclic lactones in general, 0–17% for levamisole and 0–73% for moxidectin [118]. European samples of *Cooperia* spp. and *O. ostertagi* have both shown resistance [112]. Decreased efficacy of the macrocyclic lactones ivermectin and moxidectin was reported in European farms, with confirmed anthelmintic resistance on 12.5% of these farms [119]. Continued shedding of *Cooperia* spp. and *O. ostertagi* larvae from treated cattle were identified, and this was noted particularly from farms in UK and Germany. Resistance was not detected in dairy farms in France and Italy [120]; however, grazing management interventions are proposed in these countries to limit GI nematode infestations and decrease the resistance risk.

Resistance is also an emerging problem in liver flukes [118] and bovine fasciolosis control is further limited by the limited medication options currently available against trematodes. Furthermore, treatment options available have a low efficacy against *F. hepatica* juvenile stages, a problem that exacerbates treatment resistance. However, efficacy continues to be observed; *F. hepatica* infested dairy cows in Belgium treated with Closantel 5% oral solution (0.2 ml per kg bw) before expected calving had a 305-d milk production increase (293 total L or 0.95 L of milk/d/animal) [40]. Epidemiological research on bovine fasciolosis biology and factors promoting spread is needed to improve pasture management fluke control, and limiting cattle access to wet or marshy areas that harbor snail populations is a sound strategy [43].

Development of novel treatment options continues to be essential for all types of parasites. Four approaches that support this development are: (1) screen large numbers of potential candidate molecules for antiparasite activity using methods that allow rapid screening against key parasite targets; (2) explore treatment options that introduce novel combinations of existing effective antiparasite treatments; (3) expand labels for existing approved drugs into other therapeutic indications against additional parasites; and (4) identify new bioactive plant-origin molecules, for example tannins from legumes (Fabaceae) and sesquiterpene lactones from chicory (Astareaceae) [111, 121].

Tick, *R. (B.) microplus*, control depends on effective acaricides [122] that offer relatively quick, cost-effective activity against various tick life stages. Acaricides may be administered through dips, tags, oral medications, topical pour-ons, and recently an effective injectable treatment became available. Producer education is very important for safe and effective use of these products [92]. For example, careless use of organophosphate topical products in the past led to serious health problems for administration personnel. Acaricide efficacy is more effective when knowledge of the tick life cycle and biology is used in selecting treatment timing. This approach lowers treatment costs and reduces potential impact on nontarget arthropods [123]. This strategic approach is referred to as integrated pest management and is economically efficient for dairy cattle production [124].

Problems can occur in association with acaricide use, for example resistance to selected acaricides and tick repellents are reported in some tick species [125] Monitoring of cattle after treatment for evidence of ongoing tick infestation provides an indication that an alternative acaricide class could be needed [92]. Food safety is another important consideration and acaricide milk contamination has been detected in the past. Finally, careless use of acaricides could kill beneficial species, which should be minimized through the implementation of an integrated pest management program.

There is evidence of a bovine immune response to ticks that suggests potential for future acaricide control vaccines. One vaccine tested reduced the reproductive performance of *R. (B.) microplus* ticks and helped in controlling cattle tick populations [126]. Vaccines against ticks [44] may not provide protection against all ticks but may reduce clinical impacts of tick bites and potentially decrease tick transmitted disease risks.

Using breeding and genetic selection to find cattle strains with an elevated level of tick resistance is also likely to contribute to effective long-term tick control [127]. Hemolytic analyses, measures of skin hypersensitivity reactions, and artificial tick challenges provide experimental methods for evaluating inherited cattle strain resistance to ticks [128].

### Parasite control by production type

Parasite management for dairy cattle will naturally vary among different farming systems. The goal for small producers with limited resources may be to prevent mortality, while intensive systems with many cows will aim to optimize farm productivity. Below are some considerations for parasite control in three stages of dairy production.

Dairy producers and veterinarians can implement the following steps to develop a successful parasite control program: select an effective product, treat at the optimal times for adult dairy cows, treat replacement heifers and other young stock and, maintain an annual testing and treatment program [103]. The change in environment for dairy cows that leave their grazing pasture and enter confinement housing provides an opportune time to eliminate pasture-transmitted parasites. [108]. In addition, grazed and nongrazed dairy cows should be treated prior to entering the milking string.

Estimated parasite exposure level is an important consideration in preparing the annual dairy cow treatment plans. If there is a high parasite contamination level, then treat all cows in the herd to remove parasite infections to begin the program; treat at freshening and again 6–8 weeks later. If parasite contamination is moderate, then treat all cows at freshening and again 6–8 weeks later (second treatment optional during winter.). In the case of a low estimated parasite contamination, then treat once a year at freshening and a second treatment is probably not necessary. No treatment is required if there is a negative fecal exam and where there is an extremely low parasite risk level [129].

Antiparasite treatments should be in accordance with local government requirements, for example in the USA these administrations need to follow the US Food and Drug Administration (FDA) approved label statements regarding use in dairy cows. If the cow is lactating, then any required milk withdrawal time must be followed. Treatments should be highly efficacious—> 98% efficacy against all important endoparasites (including lungworm) and all stages of parasites within the animal. For example, fenbendazole can be used at any stage of lactation or gestation, with zero milk withdrawal [130].

Antiparasite treatment may be administered to one or more individuals or to the whole herd. Individual cows can be treated every 2–3 weeks in the prefresh group, or at the time of calving. Repeated treatment at 6 weeks postpartum or at breeding time is recommended, because these are times of moderate-to-high levels of parasite exposure. It is important to treat pregnant cows for parasites just prior to freshening, to ensure they are parasite-free at the beginning of lactation. See results presented earlier showing milk production increases and favorable treatment cost recovery in cows treated at this time [17, 18, 40].

A whole herd treatment regime could be initiated in late autumn, with follow-up treatment 4–6 weeks into spring grazing. This approach keeps cattle parasite-free over the winter until the return to spring pasture. Young stock should be treated twice, 4 weeks apart, in spring to reduce pasture contamination over the summer grazing season. Cattle raised in total confinement should be parasite screened every 6 months. Antiparasitic treatment can be administered at breeding time and again just prior to entering the milk herd, i.e., just prior to freshening. Occasional ongoing fecal checks will ensure cattle remain parasite-free. Treat the whole herd if any parasites are seen [130].

Table 2 collates the key points from this paper, demonstrating the value of good dairy management as it leads to improved parasite control. This table was meant to enhance the field conversations with dairy farmers when making recommendations for parasite control.

**Table 2:** Economic insights into the impact of parasitism in dairy cattle by parasite category

Category	Impact
Parasite control in dairy cattle	1. The cost to dairy production from parasitism can be attributed to prolonged heifer rearing, reduced carcass weight, milk yield and fertility, anthelmintic treatment, and labor [13] 2. Parasite control practices can optimize productivity and financial returns [12] 3. Within a given region, no single parasite control strategy will be suited for all herds. Selection and use of anthelmintics and acaricides must be based on parasite prevalence, resistance profile, and appropriate timing of administration [12]
Nematode control	1. GI nematode infections in dairy herds cause productivity losses through reduced feed intake and decreased efficiency of its use [60], which contribute to lowered milk yield and fertility rates, increased treatment costs, delayed growth, and mortality in calves [56–58] 2. The cost of nematode infections was estimated to be between €11–87 million annually in the European Union (the main milk producer worldwide) [14], over $3 billion annually in North America [20], $7.11 billion per year in Brazil [27], and $ 445.10 million annually in Mexico [30] 3. The use of anthelmintics programs combined with pasture management practices is the ideal control practice against GI nematodes in dairy herds [18] 4. Anthelmintic treatment against nematode infections in grazing dairy herds can improve growth in calves and milk production by up to 1 kg of milk per cow per day [4, 15–17, 23] 5. Inappropriate use of anthelmintic drugs has resulted in drug-resistant nematode populations. Therefore, strategies containing two or more anthelmintics (benzimidazoles + macrocyclic lactones) are recommended as a means to slow the development of this resistance [108] 6. The fecal egg count reduction test (FECRT) is the best way for the practitioner to help producers verify that the dewormer(s) they are using is effective
Trematode control	1. Worldwide, bovine fasciolosis affects over 600 million animals and causes global economic losses of over $3 billion USD per year [6, 31–34] 2. Fasciolosis causes economic losses due to liver condemnation, decreased milk production, reduced fertility rates in cows, and incurred treatment costs [5, 35, 36] 3. Global prevalence ranges (%) of liver fluke *F. hepatica* in ruminants in different continents were: Africa 1.2–91%; South America 3–66.7%; Asia 0.7-69.2%; Australia 26–81%, and Europe 0.12–86% [34] 4. A study in India showed an increase of 4–18% in milk production after treatment with triclabendazole in dairy farms with liver fluke *F. hepatica* [6]
Tick-borne pathogens and flies	1. Ticks are responsible for many economic losses due to discomfort caused by the bite, morbidity and mortality associated with tick-borne diseases, decrease in milk production, reduction in body weight gain, and costs for application of control measures such as acaricides 2. Global economic losses from ticks and tick-borne diseases are estimated to range from $22 to $30 billion USD per annum [44] 3. In Brazilian dairy cattle, *Riphicephalus (Boophilus) microplus* was responsible for a reduction of 90.24 L of milk per cow per lactation period [28] 4. Estimated losses of babesiosis and anaplasmosis in Kenya, Zimbabwe, Tanzania, South Africa, China, India, Indonesia, and Philippines cost 5.1, 5.4, 6.8, 21.6, 19.4, 57.2, 3.1 and 0.6 million US dollars annually, respectively [46], and 16.9 million US dollars in Australian dairy cattle [45] 5. *Haematobia irritans*, *Stomoxys calcitrans*, and *Tabanus* are hematophagous flies that cause bites and discomfort with significant economic losses due to reductions in dairy production

## Conclusions

Unrecognized dairy cattle parasitism is very damaging to the global supply of milk and milk products resulting in billions of dollars in losses. The types of harm caused by unseen parasitism includes illness, death, reduced growth, lower milk production, lower reproductive success, reduced carcass quality, food safety, and animal movement restrictions. Treatment resistance, lack of treatment alternatives, and gaps in scientific knowledge and education contribute to global dairy production losses. Investment is also needed in diagnostic screening test methods and effective vaccine development. Implementing strategic parasite control measures on the basis of knowledge of prevalence, parasite biology, epidemiology of infestations, resistance profiles, and strategic grazing management continues to be critical and veterinarians and producers need to work together to improve positive economic returns. Because many therapeutic products have a withdrawal period for milk, antiparasitic treatment of lactating cows can be challenging from a human safety point of view. Dairy farmers should work with local and regional veterinarians and take note of withdrawal times of antiparasitic drugs as well as the timing of treatment regimens when putting together a treatment plan for their herds.

## Data Availability

Data supporting the main conclusions of this study are included in the manuscript.
